# Supplementary material for: A surge in respiratory syncytial virus infection-related hospitalizations associated with the COVID-19 pandemic: An observational study at pediatric emergency referral hospitals in Tokushima Prefecture
Source: PLOS Glob Public Health. 2023 Jun 2;3(6):e0001974. doi: 10.1371/journal.pgph.0001974 (PMC10237384; doi:10.1371/journal.pgph.0001974)
Supplement: S1 Table — (DOCX) [file pgph.0001974.s002.docx]

**S1 Table. Epidemiological changes in characteristics of infants aged 6-11 months**

HFNC, high-flow nasal cannula; RSV, respiratory syncytial virus; SD, standard deviation

|  | 2018–2020  (n=89) | 2021  (n=31) | *P*-value |
| --- | --- | --- | --- |
| Sex: male, n (%) | 56 (62.9) | 14 (45.2) | 0.0946 |
| Palivizumab indication, n (%)  not indicated | 2 (2.2)  87 (97.8) | 0 (0)  31 (100.0) | 1 |
| Palivizumab prophylaxis, n (%)  not administered | 1 (1.1)  88 (98.9) | 0 (0)  31 (100.0) | 1 |
| Presence of siblings, n (%)  no siblings  unknown | 46 (51.7)  19 (21.3)  24 (27.0) | 20 (64.5)  4 (12.9)  7 (22.6) | 0.284 |
| Hospitalization duration  mean days (SD) | 5.15 (1.87) | 4.81 (1.51) | 0.317 |
| Oxygen use rate, n (%) | 40 (44.9) | 9 (29.0) | 0.141 |
| HFNC use rate, n (%) | 2 (2.2) | 1 (3.2) | 1 |
| Ventilator use rate, n (%) | 0 (0) | 0 (0) | NA |
| Mortality rate, n (%) | 0 (0) | 0 (0) | NA |
