# Supplementary material for: A surge in respiratory syncytial virus infection-related hospitalizations associated with the COVID-19 pandemic: An observational study at pediatric emergency referral hospitals in Tokushima Prefecture
Source: PLOS Glob Public Health. 2023 Jun 2;3(6):e0001974. doi: 10.1371/journal.pgph.0001974 (PMC10237384; doi:10.1371/journal.pgph.0001974)
Supplement: S2 Table — (DOCX) [file pgph.0001974.s003.docx]

**S2 Table. Epidemiological changes in characteristics of infants aged 12-23 months**

HFNC, high-flow nasal cannula; RSV, respiratory syncytial virus; SD, standard deviation

|  | 2018–2020  (n=181) | 2021  (n=50) | *P*-value |
| --- | --- | --- | --- |
| Sex: male, n (%) | 103 (56.9) | 30 (60.0) | 0.748 |
| Palivizumab indication, n (%)  not indicated | 2 (1.1)  179 (98.9) | 1 (2.0)  49 (98.0) | 0.521 |
| Palivizumab prophylaxis, n (%)  not administered | 0 (0)  181 (100.0) | 0 (0)  50 (100.0) | 1 |
| Presence of siblings, n (%)  no siblings  unknown | 80 (44.2)  36 (19.9)  65 (35.9) | 28 (56.0)  10 (20.0)  12 (24.0) | 0.685 |
| Hospitalization duration  mean days (SD) | 4.97 (1.67) | 4.94 (1.82) | 0.91 |
| Oxygen use rate, n (%) | 71 (39.2) | 25 (50.0) | 0.196 |
| HFNC use rate, n (%) | 3 (1.7) | 1 (2.0) | 1 |
| Ventilator use rate, n (%) | 0 (0) | 0 (0) | NA |
| Mortality rate, n (%) | 0 (0) | 0 (0) | NA |
