# Supplementary material for: A surge in respiratory syncytial virus infection-related hospitalizations associated with the COVID-19 pandemic: An observational study at pediatric emergency referral hospitals in Tokushima Prefecture
Source: PLOS Glob Public Health. 2023 Jun 2;3(6):e0001974. doi: 10.1371/journal.pgph.0001974 (PMC10237384; doi:10.1371/journal.pgph.0001974)
Supplement: Attachment — Submitted filename: Response_to_Reviewers_20230505_0410.docx [file pgph.0001974.s004.docx]

**Response to Editor’s comments:**

1. The editor stated as follows: Please review your reference list to ensure that it is complete and correct. If you have cited papers that have been retracted, please include the rationale for doing so in the manuscript text, or remove these references and replace them with relevant current references. Any changes to the reference list should be mentioned in the rebuttal letter that accompanies your revised manuscript. If you need to cite a retracted article, indicate the article’s retracted status in the References list and also include a citation and full reference for the retraction notice.

**Response:**

Thank you for your insightful suggestion. We have reviewed the references list accordingly to ensure its completeness and correctness.

2. The editor stated as follows: We ask that a manuscript source file is provided at Revision. Please upload your manuscript file as a .doc, .docx, .rtf or .tex.

**Response:**

We have uploaded the manuscript in the .docx format accordingly.

3. The editor stated as follows: In the online submission form, you indicated that "Raw data were generated at Tokushima Prefectural Central Hospital and Tokushima Red Cross Hospital. Derived data supporting the findings of this study are available from the corresponding author KS on request". All PLOS journals now require all data underlying the findings described in their manuscript to be freely available to other researchers, either 1. In a public repository, 2. Within the manuscript itself, or 3. Uploaded as supplementary information. This policy applies to all data except where public deposition would breach compliance with the protocol approved by your research ethics board. If your data cannot be made publicly available for ethical or legal reasons (e.g., public availability would compromise patient privacy), please explain your reasons by return email and your exemption request will be escalated to the editor for approval. Your exemption request will be handled independently and will not hold up the peer review process, but will need to be resolved should your manuscript be accepted for publication. One of the Editorial team will then be in touch if there are any issues.

**Response:**

Thank you for providing us with this information. As you instructed, we have uploaded the raw data used in this study as supplementary information (S1).

Ultimately, we would like to express our sincere gratitude to the editors and reviewers for their positive and constructive criticism. The manuscript has vastly benefited from your valuable and insightful comments and suggestions. We look forward to hearing from you and would be happy to address any further concerns, if required. We hope this further pushes the manuscript closer to publication in your esteemed journal.

**Response to Reviewer #1：**

1. The reviewer gave us the following critical comments and suggestions: The first point, which I consider crucial for decision-making regarding the publication of the work, refers to the inclusion criteria for the selected patients. On page 6, line 78, the authors state, "The following eligibility criteria were included in this study: positive results on a rapid diagnostic test or clinical diagnosis of RSV infection by a physician." I understand that many paediatricians may give a clinical diagnosis of bronchiolitis, but not all these cases may be caused by RSV. The authors do not report in the results how many diagnoses were made by rapid tests and how many were made clinically. I would like the authors to clarify the criteria used for exclusive clinical diagnosis and what proportion of clinical or laboratory diagnoses were made, as I believe this could impact the results.

**Response:**

Thank you for your valuable suggestions.

First, we assessed about eligibility criteria according to your suggestion.

The “clinical diagnosis of RSV infection by a physician” has been revised as the following:

“2．clinical diagnosis of RSV infection by a physician, with an outbreak of RSV infection in the surroundings (including negative results on a rapid antigen test or without the test)” (page 6, lines 83-84).

In addition, the following sentence has been replaced to explain “exclusion criteria”:

“Cases without an outbreak of RSV infection in the surroundings and negative results on a rapid antigen test were excluded.” (page 6, lines 85-86).

Finally, we have reviewed the medical records again. Out of the total 834 cases, “positive results on a rapid antigen test” comprised 820 (98.3%) and “clinical diagnosis of RSV infection by a physician” comprised 14 (1.7%) cases.

We have added the following sentence:

“In this study, 834 cases were analyzed. Out of the total 834 cases, positive results on a rapid antigen test comprised 820 (98.3%), and clinical diagnosis of RSV infection by a physician comprised 14 (1.7%).” (page 9, lines 128-129).

2. The reviewer asked other questions about the performance of molecular tests in the diagnostic methods: Does the Tokushima prefecture perform molecular tests for patients with bronchiolitis symptoms and negative rapid tests for RSV? Has any other important etiological agent been identified? .

**Response:**

Thank you for your valuable question. We did not perform rapid molecular diagnostic test or virus isolation for RSV infections.

We have added the following sentence accordingly:

“We did not perform the rapid molecular diagnostic test or virus isolation for RSV infections.” (page 9, lines 131-132).

3．The reviewer gave us the following indication: On page 12, at the end of Table 1, the Ventilator use rate, n (%) was not statistically significant and is in bold, which needs to be fixed by removing the bold formatting.

**Response:**

We removed the bold formatting as you pointed out (Table 1).

We appreciate your meticulous indication.

4. The reviewer gave us the following comments: On page 16, in the second paragraph, the author presents data on the significant increase in COVID-19 infections in Tokushima comparing 2020 and 2021 and states that the government continues to recommend infection control measures but does not discuss what led to this increase. Tokushima went from 199 cases in 2020 to over 3000 in 2021, and this increase is very significant! Discussing some factors that led to this significant increase is essential to understand the increase in COVID-19 cases and those of RSV.

**Response:**

In 2021, Japan saw waves 3–5, with the Delta variant predominating. The COVID-19 pandemic was centered on socially active adults aged 20–59 years, with few infections among those aged 19 years and younger. It is likely that stay-homes continued to be used by families with elderly persons and families with younger children, fearing the delta strain and the resulting severe illness. However, since there was no epidemic of RSV infection in 2020, it was assumed that RSV infection spread to an unprecedented extent, especially among children aged 2 years and older who were unaffected by RSV, were not immune, and could not wear masks. The high proportion of children younger than 6 months with siblings in the survey suggested that the older children attending daycare brought RSV home and may have spread it to younger children under the influence of stay-home.

We have added the following sentence:

“In Japan, in 2021, COVID-19 was centered on socially active adults aged 20–59 years, with few infections among those aged ≤19 years [21]. People with older relatives or young children in their families were more likely to fear the severity of the disease.” (page 17, lines 205-208).

Furthermore, we added the following sentence:

“Therein, a resurgence of influenza has occurred; however, an influenza outbreak has not been observed in Japan yet, suggesting that the epidemiology of infectious diseases after the onset of COVID-19 is different in both countries.” (page 17, lines 200-202).

Ultimately, we would like to express our sincere gratitude to the editors and reviewers for their positive and constructive criticism. The manuscript has vastly benefited from your valuable and insightful comments and suggestions. We look forward to hearing from you and would be happy to address any further concerns, if required. We hope this further pushes the manuscript closer to publication in your esteemed journal.

**Response to Reviewer #2：**

1. The reviewer suggested us to give the readers additional information about location, general population and number of children under five (or older) years of age in Tokushima Prefecture.

**Response:**

Thank you for your valuable comment.

Location: Tokushima Prefecture is located in the eastern part of Shikoku island, measuring 107.37 km from east to west and 79.03 km from north to south. It is connected to Honshu island by a bridge, accessible within a day trip from Osaka city

General population: 719,559

The number of children under five years: 22,651

We have added the following sentence:

“Tokushima Prefecture is located in the eastern part of Shikoku island, measuring 107.37 km from east to west and 79.03 km from north to south. It is connected to Honshu island by a bridge, accessible within a day trip from Osaka city [12]. In October 2020, the general population in Tokushima Prefecture was 719,559 and the number of children aged under five years was 22,651 [13].” (page 6, lines 89-92).

2. The reviewer pointed out about type of access to the hospitals included in the study (public or private funding).

**Response:**

Thank you for pointing this out.

Tokushima Prefectural Central Hospital: public funding

Tokushima Red Cross Hospital: public funding

We have added the following sentence:

“Children who were admitted to the Department of Pediatrics at Tokushima Prefectural Central Hospital (public funding) and the Department of Pediatrics at Tokushima Red Cross Hospital (public funding), in Tokushima Prefecture,” (page 5, lines 76-78).

3. The reviewer asked whether nominal or non-nominal identification of electronic medical records.

**Response:**

Thank you for your valuable query.

Data were extracted from nominal identification of electronic medical records and entered into the database as non-nominal identification.

We have added the following sentences:

“data were extracted via nominal identification of electronic medical records of children” (page 5, lines 74-75).

“Data on the following variables were extracted and reviewed, as far back as possible, from the non-nominal identification databases of the participating hospitals” (page 7, lines 103-104).

4. The reviewer pointed out about maximum age of children included in the study.

**Response:**

Thank you for pointing this out.

The maximum age of children was 172 months.

We have uploaded the raw data so that the age distribution is better presented. (S1)

We have added the following sentence:

“The maximum age of children was 172 months.” (page 9, line 132).

5. The reviewer commented whether there were hospitalizations for non-resident children in Tokushima Prefecture at the reference hospitals during the study time.

**Response:**

Thank you for your pertinent comment.

We conducted an additional survey.

We have added the following sentence accordingly:

“Among the 834 cases, 813 (97.5%) were residents of Tokushima Prefecture, whereas 21 (2.5%) were non-residents.” (page 9, lines 132-133).

6. The reviewer asked whether there was reallocation of pediatric care to the reference hospitals in the study during the periods before and after the COVID-19 pandemic.

**Response:**

Thank you for your valuable comment. There was no reallocation of pediatric care to the referral hospitals in the study during the periods.

We have added the following sentence:

“In the study, there was no reallocation of pediatric care to the reference hospitals during this period.” (page 7, lines 100-101).

7. The reviewer gave us following questions and suggestions: Did the electronic medical records allow for long-term follow-up of children (medical history)? Or, were the data about the variables obtaining only at the time of hospitalization for RSV? My question is fundamented in terms of the proportion of children ≥24 months of age with an unknown status regarding having received, palivizumab prophylaxis in comparison with children <6 months of age (estatistical significance). Could there be a memory bias regarding answering this question during hospitalization? Maybe, the information about the time and the measure procedures of the variable ", palivizumab prophylaxis" could offer more support for the discussion text about the differences of proportion.

**Response:**

As for palivizumab prophylaxis, we determined from the electronic medical records whether or not it was indicated with respect to that season. Thus, it seems that there was not much memory bias, but there may be some interview bias on the part of the healthcare provider. Thank you for your valuable suggestions. In addition, as you mentioned in your question, the electronic medical record can allow for long-term follow-up, and we have reviewed as far back as possible from the electronic medical record whether or not palivizumab was indicated at the season in which the patient was admitted to the hospital. According to your suggestion, we made the following correction.

We have added the following sentences:

“Data on the following variables were extracted and reviewed as far back as possible from the non-nominal identification databases of the participating hospitals” (page 7, lines 103-104)

“palivizumab indication (at the season), palivizumab prophylaxis (at the season)” (page 7, lines 105-106).

Thank you for your valuable suggestions, “Unknown” has been eliminated; we now have clearer data. We have also added a note to the discussion section.

We have added the following sentence:

“In 2021, there was no significant change in both palivizumab indication and palivizumab prophylaxis among hospitalized children.” (page 18, lines 219-220).

8. The reviewer gave us the following questions: Was there difference in the guidelines for rapid diagnostic testing and clinical diagnosis of RSV between the before and after periods? Was there a possibility of incorrect classification of the clinical diagnosis for other respiratory infections? Viral isolation was a widely used method for the diagnosis of RSV infection in the reference hospitals during the period of the study?

**Response:**

Thank you for your invaluable inquiry.

First, there was no difference in the guidelines for rapid diagnostic testing and clinical diagnosis of RSV infection between the periods.

We have added the following sentence:

“There was no difference in the guidelines for rapid diagnostic testing and clinical diagnosis of RSV infection between the periods.” (page 9, lines 129-131).

Second, we apologize for our inadequate explanation of the diagnosis.

To clearly explain eligibility criteria, we have revised and explained by replacing with the following sentences:

“and who met the following eligibility criteria (1 or 2) were included in this study: entry in the electronic medical record as any of the following – “RSV infection,” “RSV bronchiolitis,” “RSV pneumonia,” and “RSV encephalopathy”; and treated on site during the study period, as evidenced by medical records.

1．positive results on a rapid antigen test

2．clinical diagnosis of RSV infection by a physician, with an outbreak of RSV infection in the surroundings (including negative results on a rapid antigen test or without the test)

Cases without an outbreak of RSV infection in the surroundings and negative results on a rapid antigen test were excluded.” (page 6, lines 78-86).

Third, we did not perform virus isolation for RSV infections.

We have added the following sentence:

“We did not perform the rapid molecular diagnostic test or virus isolation for RSV infections.” (page 9, lines 131-132).

Finally, the following sentence has been added as a limitation:

“Although clinical diagnoses accounted for a minority of cases (1.7% of the total), there is a possibility of incorrect classification due to potential overlap with other respiratory infections and co-infections, as rapid molecular diagnostic test or virus isolation was not performed” (page 18, lines 224-227).

9. The reviewer suggested to include tables of epidemiological changes in the characteristics of infants aged 6 to 11 and 12 to 23 months as a supplementary file.

**Response:**

Thank you for your insightful suggestion.

We included tables of epidemiological changes in the characteristics of infants aged 6 to 11 and 12 to 23 months as supplementary files accordingly (S2 and S3).

Ultimately, we would like to express our sincere gratitude to the editors and reviewers for their positive and constructive criticism. The manuscript has vastly benefited from your valuable and insightful comments and suggestions. We look forward to hearing from you and would be happy to address any further concerns, if required. We hope this further pushes the manuscript closer to publication in your esteemed journal.

**A LIST OF CHANGES MADE IN THE MANUSCRIPT**

Materials and Methods:

1. We collected “Materials and Methods” (page 5, line 71)
2. We added the following sentences: “nominal identification of” (page 5, line 74)
3. We changed the sentence “Children who were admitted to the Department of Pediatrics at Tokushima Prefectural Central Hospital (public funding) and the Department of Pediatrics at Tokushima Red Cross Hospital (public funding), in” (page 5, lines 76-77)
4. We changed the sentence “and who met the following eligibility criteria (1 or 2) were included in this study:“(page 6, line 78-79)
5. We changed the sentence “1．positive results on a rapid antigen test

2．clinical diagnosis of RSV infection by a physician, with an outbreak of RSV infection in the surroundings (including negative results on a rapid antigen test or without the test)

Cases without an outbreak of RSV infection in the surroundings and negative results on a rapid antigen test were excluded.” (page 6, lines 82-86)

6. We added the following sentence “Tokushima Prefecture is located in the eastern part of Shikoku island, measuring 107.37 km from east to west and 79.03 km from north to south. It is connected to Honshu island by a bridge, accessible within a day trip from Osaka city [12]. In October 2020, the general population in Tokushima Prefecture was 719,559 and the number of children aged under five years was 22,651 [13].” (page 6, lines 89-92)
7. We added the following sentence “In the study, there was no reallocation of pediatric care to the reference hospitals during this period.” (page 7, line 100-101)
8. We added the following phrase “and reviewed as far back as possible” (page 7, line 103)
9. We added the following phrase “non-nominal identification” (page 7, line 104)
10. We collected the following phrase “palivizumab indication (at the season), palivizumab prophylaxis (at the season)” (page 7, lines 105-106)

Results

11. We added the following sentence “In this study, 834 cases were analyzed. Out of the total 834 cases, positive results on a rapid antigen test comprised 820 (98.3%), and clinical diagnosis of RSV infection by a physician comprised 14 (1.7%). There was no difference in the guidelines for rapid diagnostic testing and clinical diagnosis of RSV infection between the periods. We did not perform the rapid molecular diagnostic test or virus isolation for RSV infections. The maximum age of children was 172 months. Among the 834 cases, 813 (97.5%) were residents of Tokushima Prefecture, whereas 21 (2.5%) were non-residents.” (page 9, line 128-133)
12. We collected the word “was” (page 11, line 158)

Discussion

13. We added the following sentence “Therein, a resurgence of influenza has occurred; however, an influenza outbreak has not been observed in Japan yet [16], suggesting that the epidemiology of infectious diseases after the onset of COVID-19 is different in both countries.” (page 17, lines 200-202)
14. We added the following sentence “In Japan, in 2021, COVID-19 was centered on socially active adults aged 20–59 years, with few infections among those aged ≤19 years [21]. People with older relatives or young children in their families were more likely to fear the severity of the disease.” (page 17, line 205-208)
15. We changed the phrase “To date” (page 17, line 208)
16. We changed the following sentence “In 2021, there was no significant change in both palivizumab indication and palivizumab prophylaxis among hospitalized children.” (page 18, lines 219-220)
17. We changed the word “some” (page 18, line 224)
18. We added the following sentence “Although clinical diagnoses accounted for a minority of cases (1.7% of the total), there is a possibility of incorrect classification due to potential overlap with other respiratory infections and co-infections, as rapid molecular diagnostic test or viral isolation was not performed.” (page 18, line 224-227)

Tables:

15. We changed the numbers in Table 1 as follows:

Palivizumab indication n (%) in 2018, 2019, 2020 and 2021, P-value

We changed the phrase “not indicated”

Palivizumab prophylaxis n (%) in 2018, 2019, 2020 and 2021, P-value

We changed the phrase “not administered”

We changed the phrase “no siblings”

We removed the bold formatting at P-value in Ventilator use rate

16. We changed the numbers in Table 2 as follows:

Palivizumab indication n (%) in 2018-2020 and 2021, P-value

We changed the phrase “not indicated”

Palivizumab prophylaxis n (%) in 2018-2020 and 2021, P-value

We changed the phrase “not administered”

We changed the phrase “no siblings”

We collected the P-value in Hospitalization duration “0.316”

17. We changed the numbers in Table 3 as follows:

Palivizumab indication n (%) in 2018-2020 and 2021, P-value

We changed the phrase “not indicated”

Palivizumab prophylaxis n (%) in 2018-2020 and 2021

We changed the phrase “not administered”

We changed the phrase “no siblings”

We collected the P-value in Hospitalization duration “0.431”

References:

25. We added the following references

12. Tokushima Tourist Information Site Awa Navi [Internet] [Cited 29 April 2023]. Available from:

https://www.awanavi.jp/document/24666.html

13. e-Stat Portal Site of Official Statistics of Japan Statistics of Japan [Internet] [Cited 29 April 2023]. Available from:

https://www.e-stat.go.jp/stat-search/files?page=1&layout=datalist&toukei=00200521&tstat=000001136464&cycle=0&year=20200&month=24101210&tclass1=000001136466We changed the expression of online material.

Supporting information:

26.	S1 Raw data in this study

27.	S2 Table4. Epidemiological changes in characteristics of infants aged 6-11 months

28.	S3 Table5. Epidemiological changes in characteristics of infants aged 12-23 months
